# Salivary cotinine level and treatment response in muscle invasive bladder cancer: A pilot study

**DOI:** 10.1002/bco2.204

**Published:** 2022-12-28

**Authors:** Jeromy W. Gotschall, C. Kendall Major, Linda A. Jacobs, Abigail Blauch, Donna Pucci, Steven C. Palmer, Ronac Mamtani

**Affiliations:** ^1^ Abramson Cancer Center University of Pennsylvania Philadelphia Pennsylvania USA

**Keywords:** biochemical marker, bladder cancer, cotinine, cystectomy, muscle invasive bladder cancer, neoadjuvant chemotherapy, platinum‐based chemotherapy, salivary cotinine, smoking, treatment efficacy

Dear Editor

Cigarette smoking is one of the most common modifiable risk factors for developing bladder cancer, with a population attributable risk of 50%–65% in men and 20%–30% in women. Bladder cancer arising in patients with tobacco exposure is on average more aggressive and has poorer outcomes.^1^ Continued tobacco use during cancer treatment can lower response rates, increase risk of recurrence, and increase overall and cancer specific mortality,^1^ potentially through alteration of tumour cell apoptosis, modification of drug delivery and metabolism, and increase in clearance of therapeutic agents.^2^ However, prior studies examining associations between smoking and muscle‐invasive bladder cancer (MIBC) treatment were limited by reliance on self‐reported smoking status, which is subject to recall bias and underreporting due to feelings of guilt or shame,^3^ potentially under‐estimating the association and risking bias towards the null.

To address the limitation of self‐reported smoking status, we undertook a pilot study employing a novel biochemical approach to assess active smoking status in patients with MIBC undergoing neoadjuvant chemotherapy. Cotinine is the central metabolite of nicotine and can be detected in saliva for up to 4 days after tobacco use.^4^ Thus, salivary cotinine concentration is a reliable surrogate for recent tobacco use. The NicAlert testing system is a semi‐quantitative tool that provides categorical data on salivary cotinine concentration, with increasing levels corresponding to greater tobacco use.^5^

In this prospective cohort study, our objectives were to (i) compare cotinine levels to self‐reported smoking status, and (ii) to compare pathologic response at the time of cystectomy in smokers vs non‐smokers based on cotinine level.

Thirty‐one patients with MIBC initiating neoadjuvant chemotherapy were prospectively recruited and surveyed on their smoking habits. Salivary cotinine was measured during chemotherapy using NicAlert test strips, where 0 corresponds to a cotinine concentration <10 ng/ml, indicating no active smoking, and 1–6 corresponds to increasing concentrations of cotinine, indicating active tobacco use. The primary outcome was pathologic response at cystectomy, defined as complete response (pT0) or residual disease (pT1–4 or node+). The primary exposure was active smoking or no active smoking, defined using salivary cotinine concentration level (<10 and >10 ng/ml, respectively).^5^ The association between pre‐chemotherapy cotinine level and post‐chemotherapy response at cystectomy was assessed using logistic regression models.

Thirty‐one patients had cotinine levels measured during the time in which they received neoadjuvant chemotherapy. Twenty‐five participants endorsed a prior history of smoking, 18 of whom denied current active smoking. Of these 18 patients, 2 were found to have cotinine levels >10 ng/ml during chemotherapy. Among the 7 patients who reported active smoking, 1 was found to have cotinine <10 ng/ml during chemotherapy. Thus, biochemical evidence of tobacco use differed from self‐reported smoking status in 3 of 25 patients (12%). One patient declined cystectomy and was excluded from further analyses.

Response to chemotherapy among patients with prior history of tobacco use is shown in Table 1, comparing patients with cotinine concentrations <10 ng/ml versus >10 ng/ml. Rates of complete pathologic response were numerically higher for patients with cotinine <10 ng/ml (56%) versus cotinine >10 ng/ml (25%), corresponding to an odds ratio of 3.86 (95% CI: 0.54–22.05, *p* = 0.148). Likewise, the rates of residual disease were much lower for those with cotinine concentrations <10 ng/ml (44%) versus cotinine >10 ng/ml (75%), OR, 0.26; 95% CI: 0.045–1.86, *p* = 0.148.

**TABLE 1:** Odds of complete response vs. residual disease by cotinine level

	Cotinine <10 ng/ml ‘non‐active tobacco use’		Cotinine >10 ng/ml ‘active tobacco use’		
	*N*	Probability	*N*	Probability	Odds ratio
Complete response (pT0)	9	0.56	2	0.25	3.86
Residual disease (pTis, T1, T2, T3, T4)	7	0.44	6	0.75	0.26
Total	16	1.00	8	1.00	

In this pilot study, we demonstrate that prior smokers with no biochemical evidence of active tobacco use during curative chemotherapy had more than three‐times greater odds of demonstrating a complete response at the time of cystectomy compared to those with evidence of current tobacco use based on salivary cotinine levels, although these findings did not achieve statistical significance. While this study was limited by a small sample size, it demonstrates a feasible approach to tobacco use monitoring and the importance of continued smoking cessation counselling in patients with bladder cancer even after the diagnosis of cancer is made. Approximately 10% of the cohort (2/18) denied active smoking yet were found to have biochemical evidence of current tobacco use/exposure, implying cotinine monitoring helped to reduce self‐report bias. Indeed, using self‐report data alone, our study would have underestimated the relative odds of poor response to chemotherapy among active smokers by over 25% (OR of 2.81 vs. 3.86). Furthermore, this study identifies a subgroup of patients, that is, active smokers, who may have disease resistant to standard cisplatin therapy and thus may benefit from alternative therapeutic approaches such as upfront surgery or neoadjuvant immunotherapy.^6^ These data support integration of automated referrals for patients who smoke to evidence‐based tobacco treatments following models that leverage implementation science and clinical informatics to improve clinical outcomes.^7^

The main limitation of our study is the small sample size. An additional limitation is residual confounding due to passive smoking (i.e., exposure to second‐hand smoke in a patient's living situation) and other non‐smoking nicotine exposures such as nicotine replacement therapy, smokeless tobacco, and e‐cigarettes, which were not measured in our study. Indeed, passive smokers and those using other nicotine products could present with elevated cotinine levels and may show varied responses to chemotherapy. We recommend that future studies assess for both active and passive smoking along with exposure to nicotine products to ensure appropriate interpretation of cotinine levels. Another limitation to this study is the short half‐life of cotinine and variability due to genetic, hormonal, and other factors, leading to potential misclassification of active tobacco users. For example, one patient in our study who self‐reported active tobacco use was found to have cotinine concentration <10 ng/ml during therapy. To overcome this limitation, future studies should implement more frequent cotinine monitoring intervals, such as every 4 days while undergoing active therapy. Lastly, our analysis was not powered to assess for a dose response between increasing cotinine concentrations and poorer pathologic response rates. This would delineate if reducing smoking, when unable or unwilling to quit entirely, and would still benefit patients with MIBC undergoing neoadjuvant chemotherapy.

In conclusion, our study demonstrates a detrimental effect of active smoking on treatment response among patients with MIBC receiving curative chemotherapy and supports saliva‐based cotinine monitoring as an innovative strategy to address self‐report bias in studies investigating the effects of recent tobacco exposure on treatment outcomes for this population. Unfortunately, tobacco use assessments in this setting are rare and omitted from current clinical practice guidelines.^8^

## CONFLICTS OF INTEREST

Dr. Mamtani reported receiving honoraria from Flatiron Health and research funding from Merck. He also served in an advisory role for Genetech/Roche and Seattle Genetics/Astellas. No other disclosures were reported.

## AUTHOR CONTRIBUTIONS

Study conception and design: RM, LAJ, AB, DP, SCP; data collection: RM, LAJ, AB, DP, SCP; analysis and interpretation of results: RM, JWG, CKM; draft manuscript preparation: JWG, CKM. All authors reviewed the results and approved the final version of the manuscript.
